# Identification of Crucial Genes and Key Functions in Type 2 Diabetic Hearts by Bioinformatic Analysis

**DOI:** 10.3389/fendo.2022.801260

**Published:** 2022-02-15

**Authors:** Xin Huang, Kai-jie Zhang, Jun-jie Jiang, Shou-yin Jiang, Jia-bin Lin, Yi-jia Lou

**Affiliations:** ^1^ Cardiovascular Key Laboratory of Zhejiang Province, The 2nd Affiliated Hospital, School of Medicine, Zhejiang University, Hangzhou, China; ^2^ Biotherapy Research Center, The 2nd Affiliated Hospital, School of Medicine, Zhejiang University, Hangzhou, China; ^3^ Institute of Pharmacology and Toxicology, College of Pharmaceutical Sciences, Zhejiang University, Hangzhou, China; ^4^ Chu Kochen Honors College, Zhejiang University, Hangzhou, China; ^5^ Department of Emergency Medicine, The 2nd Affiliated Hospital, School of Medicine, Zhejiang University, Hangzhou, China; ^6^ Clinical Research Center, The 2nd Affiliated Hospital, School of Medicine, Zhejiang University, Hangzhou, China

**Keywords:** type 2 diabetes, acute cardiac injury, differentially expressed genes, bioinformatics, calpain small subunit 1 (capn4), COVID-19, self-protective role

## Abstract

Type 2 diabetes (T2D) patients with SARS-CoV-2 infection hospitalized develop an acute cardiovascular syndrome. It is urgent to elucidate underlying mechanisms associated with the acute cardiac injury in T2D hearts. We performed bioinformatic analysis on the expression profiles of public datasets to identify the pathogenic and prognostic genes in T2D hearts. Cardiac RNA-sequencing datasets from *db/db* or BKS mice (GSE161931) were updated to NCBI-Gene Expression Omnibus (NCBI-GEO), and used for the transcriptomics analyses with public datasets from NCBI-GEO of autopsy heart specimens with COVID-19 (5/6 with T2D, GSE150316), or dead healthy persons (GSE133054). Differentially expressed genes (DEGs) and overlapping homologous DEGs among the three datasets were identified using DESeq2. Gene Ontology (GO) and Kyoto Encyclopedia of Genes and Genomes analyses were conducted for event enrichment through clusterProfile. The protein-protein interaction (PPI) network of DEGs was established and visualized by Cytoscape. The transcriptions and functions of crucial genes were further validated in *db/db* hearts. In total, 542 up-regulated and 485 down-regulated DEGs in mice, and 811 up-regulated and 1399 down-regulated DEGs in human were identified, respectively. There were 74 overlapping homologous DEGs among all datasets. Mitochondria inner membrane and serine-type endopeptidase activity were further identified as the top-10 GO events for overlapping DEGs. Cardiac *CAPNS1* (calpain small subunit 1) was the unique crucial gene shared by both enriched events. Its transcriptional level significantly increased in T2D mice, but surprisingly decreased in T2D patients with SARS-CoV-2 infection. PPI network was constructed with 30 interactions in overlapping DEGs, including *CAPNS1*. The substrates *Junctophilin2* (*Jp2*), *Tnni3*, and *Mybpc3* in cardiac calpain/CAPNS1 pathway showed less transcriptional change, although *Capns1* increased in transcription in *db/db* mice. Instead, cytoplasmic JP2 significantly reduced and its hydrolyzed product JP2NT exhibited nuclear translocation in myocardium. This study suggests *CAPNS1* is a crucial gene in T2D hearts. Its transcriptional upregulation leads to calpain/CAPNS1-associated JP2 hydrolysis and JP2NT nuclear translocation. Therefore, attenuated cardiac *CAPNS1* transcription in T2D patients with SARS-CoV-2 infection highlights a novel target in adverse prognostics and comprehensive therapy. CAPNS1 can also be explored for the molecular signaling involving the onset, progression and prognostic in T2D patients with SARS-CoV-2 infection.

## Introduction

Type 2 diabetes (T2D) as a chronic medical condition is one of the most important risk factors for adverse outcomes of coronavirus disease 2019 (COVID-19) caused by severe acute respiratory syndrome coronavirus 2 (SARS-CoV-2) (1–4). The available data has supported that increased susceptibility in patients with T2D to SARS-CoV-2 hospitalizations (5, 6). Moreover, an acute cardiovascular manifestation of COVID-19 often presents as an acute cardiac injury with cardiomyopathy, ventricular arrhythmias, hemodynamic instability and cardiogenic shock, in the absence of obstructive coronary artery disease (7, 8). Putative mechanisms contributing to increased susceptibility for COVID-19 in patients with T2D have been concluded (3, 5, 9). The initial step is that SARS-CoV-2 binds to angiotensin-converting enzyme 2 (ACE2) expressing in key metabolic organs and tissues (10). SARS-CoV-2 directly infects cardiomyocytes *in vitro* or in T2D patients in an ACE2-dependent manner (6, 11). ACE2 cascade also associates with diabetic cardiomyopathy in *db/db* T2D mice either (12). Furthermore, SARS-CoV-2 may cause pleiotropic alterations of glucose metabolism that could complicate the pathophysiology of preexisting diabetes or lead to new mechanisms of disease (10). That means the increased susceptibility of hearts themselves in T2D patients is directly involved in the adverse outcomes of COVID-19, although SARS-CoV-2 infection alone could also cause clotting issues in the coronaries. Therefore, acute cardiac injury in T2D appears as one of the leading causes of severe disease and death in patients with COVID-19 (4, 9). To date, the significance of important mediating mechanism for the acute COVID-19 cardiovascular syndrome in T2D patients has not been fully illuminated.

Genome-wide molecular profiling is able to reveal molecular changes in disease occurrence and progression and has proved to be a high-efficient way to identify key genes (13–15). Recently, a transcriptomic analyses has been selected to identify the synergistic effect of SARS-CoV-2 infection and idiopathic pulmonary fibrosis patients in lung epithelium cell datasets (16, 17). Meanwhile, autopsy specimens from the patients with SARS-CoV-2 infection have also been analyzed using a combination of different RNA and protein analytical platforms to characterize inter-patient and intra-patient heterogeneity of pulmonary virus infection (18).

Given that both animal and human with diabetic cardiomyopathy share the same potential biomarkers (19), in the present study, we employ the transcriptomics analyses and key gene validation for both *db*/*db* T2D mice *vs* control (GSE161931) and autopsy specimens of T2D patients with SARS-CoV-2 infection (GSE150316) *vs* control (GSE133054) from NCBI-Gene Expression Omnibus (NCBI-GEO) (20, 21). We identify differentially expressed genes (DEGs) to characterize the overlapping homologous DEGs (16, 22). And also perform functional enrichment analyses of DEGs, identify crucial genes and validate molecular mechanism in T2D hearts, respectively (16). Our results reveal that attenuated cardiac *CAPNS1* transcription in T2D patients who succumbed to SARS-CoV-2 infection highlights a novel event in adverse prognostics, and provide for a more detailed molecular mechanism underlying the acute cardiac injury of occurrence and progression in T2D patients with SARS-CoV-2 infection. Therefore, cardiac CAPNS1 can be explored for the molecular signaling involving the onset, progression and prognostic in T2D patients with SARS-CoV-2 infection and holding promise for using as a biomarker and potential therapeutic target in anti-SARS-CoV-2 comprehensive therapy.

## Materials and Methods

### Dataset and Identification of DEGs

The gene expression profile datasets of heart samples (GSE161931) from 7 *db/db* T2D mice and 9 BKS mice as wild-type control were sequenced through Illumina NextSeq 500 platform (Novogene, Tianjing, China), and updated to NCBI-GEO in the present study (https://www.ncbi.nlm.nih.gov/geo/) (20, 21). The profile datasets of cardiac gene expression from 6 autopsy specimens patients (5/6 with T2D) who succumbed to SARS-CoV-2 infection (GSE150316), and 8 autopsy specimens from dead healthy persons (free from any major disease) (GSE133054) were obtained from GEO (https://www.ncbi.nlm.nih.gov/geo/) (20, 21, 23). The age range distributed from 30-years-old to 80-years-old of T2D patients with COVID-19 (18). Identification of DEGs was performed using DESeq2 packages based on the R programming language (version 3.12). Since R package can assist in the process of generating publication quality figures of DGE results files from *Cuffdiff*, *DESeq2* and *edger*, in order to integrate of these functions in a user-friendly way (22). Batch effect between GSE150316 and GSE133054 was removed by R package sva (version 3.38.0). The adjusted *P*-values (adj *P*-value) were adopted to avoid the occurrence of false-positive results (14). Genes with fold change (FC) larger than1.5 or less than 1/1.5 (24) and *P*-value less than 0.05 were taken as DEGs between T2D hearts and control samples. The datasets were examined both intra-groups and inter-groups to obtain overlapping DEGs and subsequently identify the genes unique to T2D hearts. Ggplot2 and Venn Diagram packages of R were applied to generate volcano plot and Venn diagram, for the visualization of identified DEGs and overlapping homologous DEGs, respectively (14).

### Functional Enrichment Analysis

Gene Ontology (GO) function (25) and Kyoto Encyclopedia of Genes and Genomes (KEGG) pathway enrichment analyses of the candidate DEGs were performed through clusterProfiler package (versions3.0.4) (14). GO or KEGG analyses were presented in dot or ridge plot format, respectively.

### Protein-Protein Interaction Network Construction and Module Analysis

The DEG-encoded proteins and their interactions amongst each other were established through the Retrieval of Interacting Genes Database (STRING, version 11.5; https://string-db.org/cgi/input.pl), visualized by Cytoscape software and further analyzed by Molecular Complex Detection (MCODE) algorithm (26, 27). Subsequently, the PPI network for overlapping homologous DEGs was constructed with a confidence score ≥ 0.7. The advanced options set as degree cut-off = 2, K-Core = 2, and Node Score Cut-off = 0.2.

### Animals and Tissue Samples

Eight male LepR*^db/db^* (*db/db)* T2D mice (eight-weeks-old) and 11 male C57BLKS/J (BKS) mice (eight-weeks-old) were purchased from Institute of Biomedical Research, Nanjing University (Nanjing, Jiangsu, China). Mice were housed in a suitable environment (23 ± 1°C and 70% humidity) with a 12-hour light-dark cycle and had free access to water and standard chow food for further 8 weeks. All studies were in accordance with the Guide for the Care and Use of Laboratory Animals (National Institutes of Health, USA) and approved by the Animal Care and Ethics Committee of Zhejiang University (№ ZJU-2015-435-01), and the entire *in vivo* study protocols were approved by the 2nd Affiliated Hospital Research Ethics Committee of Zhejiang University, China (Protocol #2020-1151). Heart tissues were obtained from mice (16-weeks-old). Briefly, mice were anaesthetized using 10% chloral hydrate. The whole heart was removed immediately, frozen with liquid nitrogen and stored at −80°C for further study. Tissue sections were prepared as reported (28), and cut into 5-μm continuous sections. For scanning electron microscope (SEM), the apex area of heart was fixed in chilled 2.5% glutaraldehyde and preceded to following steps.

### Quantitative Real-Time RT-PCR

Total RNA was extracted from heart tissues by Trizol reagent (Invitrogen). Then, 2 μg of RNA underwent reverse transcription using Reverse Transcription (RT) kit (TAKARA, Dalian, China) according to the manufacturer’s instructions. Amplifications of cDNA were performed using SYBR premix extaq kit (TAKARA) in Bio-Rad CFX 96 (Bio-Rad, California, USA). The forward and reverse primers were as shown in **Supplementary Table 1**. Measurement was normalized to *Gapdh* for *Capns1*, *Jp2* (*Junctophilin2*), *Tnni3* (troponin 1 type 3), and *Mybpc3*. The relative gene expression was presented by comparative CT method.

### Western Blot Analysis

Western blot was performed as previously described (29). Briefly, sample lysates were resolved in SDS-PAGE and transferred onto PVDF membrane (Merck Millipore, Billerica, MA, USA). The PVDF membranes were incubated with the primary antibody JP2 (ab110056, Abcam, MA, USA, 1:1000) and GAPDH (HA-ET1702-66-200, HUABIO, Hangzhou, China), respectively. Then samples were incubated with HRP-conjugated secondary antibodies (LK-GAM007, LK-GAR007, LK-RAG007, MULTISCIENCES, Shanghai, China). Blots were developed using enhanced chemiluminescence reagents (Cat#. 32106, Pierce™, IL, USA).

### Analysis of Cardiac Ultrastructure

Left ventricular tissue was dissected into 3-mm^3^ pieces and fixed in 4% glutaraldehyde and 1% osmic acid in turn. The samples were dehydrated by ascending concentrations of acetone, embedded with EPON812, stained with toluidine blue, cut into slices of 70 nm and double-stained with uranyl acetate and lead citrate. Ultrastructure was examined by transmission electron microscopy (TEM) (H7500TEM, Hitachi, Japan). The ultrastructures of mitochondria and myocardium were assessed, respectively. The visible image of 5 randomly selected areas per slice was photographed at 30000× magnification.

### Echocardiography Analysis

Mice were anaesthetized with isoflurane (1–2% in oxygen gas mixture). After shaving hair carefully on the left chest, cardiac geometry was measured from the parasternal long axis view using a small animal color ultrasonic diagnostic apparatus (Visual Sonic Vevo 2100, Toronto, ON, Canada) with a probe frequency of 30 MHz. A clear image of the left ventricular area was recorded using M-type echocardiography. Left ventricular ejection fraction (LVEF) and left ventricular fraction shortening (LVFS) were then calculated based on the mean values from 6 cardiac cycles. All the echocardiographic images were analyzed using Vevo 2100 software.

### Immunofluorescence Image Analysis

All immunostaining was conducted as previously described (29). Briefly, samples were probed with the primary antibodies as follows: cardiac troponin T (cTnT, ab8295, Abcam, MA, USA.1:100), JP2 and its N-terminal fragment (NT-JP2) (ab110056, Abcam, MA, USA, 1:100). Then samples were incubated with DyLight488 goat anti-mouse secondary antibody (GAM4882, Multisciences, (Lianke) Biotech Co., Ltd. Hangzhou, China.1:200) and 4′, 6-Diamidino-2-phenylindoledihy-drochloride (DAPI, ab188804, Abcam, MA, USA. 2 μg/ml). Images were acquired under laser scanning Confocal microscopes from Leica Microsystems (Leica Microsystems Inc., Buffalo Grove, IL, USA). Relative ratio of JP2-NT nuclear translocation was analyzed by ImageJ software (National Institutes of Health, USA).

### Statistical Analysis

Data were reported as means ± SD. Statistical analysis was performed through GraphPad Prism (version 8, San Diego, CA) software. Student’s *t*-tests were utilized for the comparison of two sample groups. Differences were considered as statistically significant when *P <*0.05.

## Results

### Identification of DEGs in T2D Hearts

**Figure 1:**
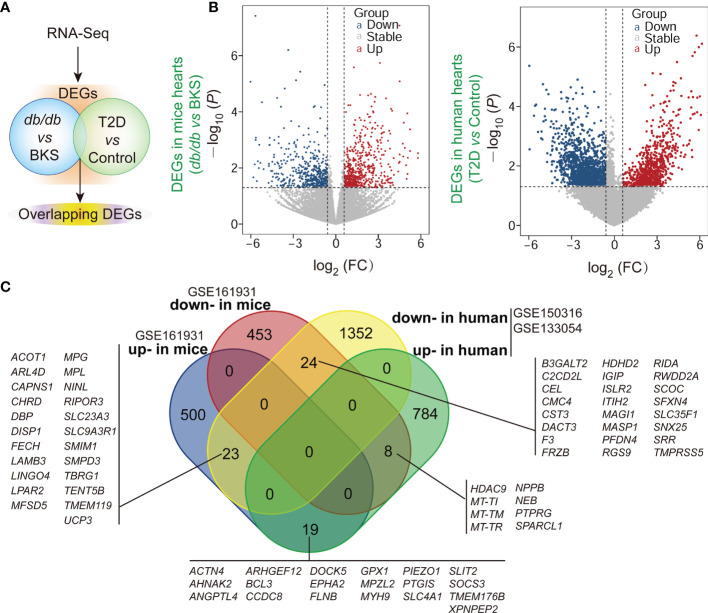
∣ Identification of differentially expressed genes (DEGs) and overlapped homologous DEGs in T2D or non-T2D hearts with or without SARS-CoV-2 infection. **(A)** Scheme of the experimental procedure. The gene expression profiles in public datasets from NCBI-GEO were analyzed to identify DEGs in the heart tissues between T2D mice and control, as well as T2D patients who succumbed to SARS-CoV-2 infection and control. **(B)** Respective volcano plots of sifted out DEGs for datasets in the accordance with public database GSE161931(mice), GSE150316 *vs* GSE133054 (human) of NCBI-GEO. Blue and red plot represent up- and down-regulated genes, respectively. Gray plot represent the remaining genes with no significant difference. **(C)** The Venn diagrams of the overlapping homologous DEGs among the three datasets. Threshold was set to be FC > 1.5 (or <1/1.5) and *P* < 0.05.

The gene expression profiles in public datasets from NCBI-GEO were analyzed to identify DEGs in the heart tissues between T2D mice and control, as well as T2D patients who succumbed to SARS-CoV-2 infection (age range from 30-years-old to 80-years-old) (18) and control (**Figure 1A**). Upon setting the cut-off criterion as genes with FC > 1.5 (or <1/1.5) and *P* < 0.05, we identified 1027 DEGs (542 up-regulated and 485 down-regulated) in GSE161931, and 2210 DEGs (811 up-regulated and 1399 down-regulated) in GSE150316 *vs* GSE133054 showed by volcano plots, respectively (**Figure 1B** and **Supplementary Tables 2**, **3**). The overlapping homologous DEGs among the three datasets were further identified. Venn diagram made all the 74 overlapping DEGs visualization (**Figure 1C** and **Supplementary Table 4**), including 23 down-regulated in T2D patients but up-regulated in T2D mice, 24 down-regulated in both patients and mice, 8 up-regulated in patients but down-regulated in mice, and 19 up-regulated in both patients and mice. These results indicated that SARS-CoV-2 infection did affect the transcriptions of some genes in T2D hearts. The overall statistics are shown in **Supplementary Tables 2**, **3**.

### Functional Enrichment and Module Analysis of DEGs

**Figure 2:**
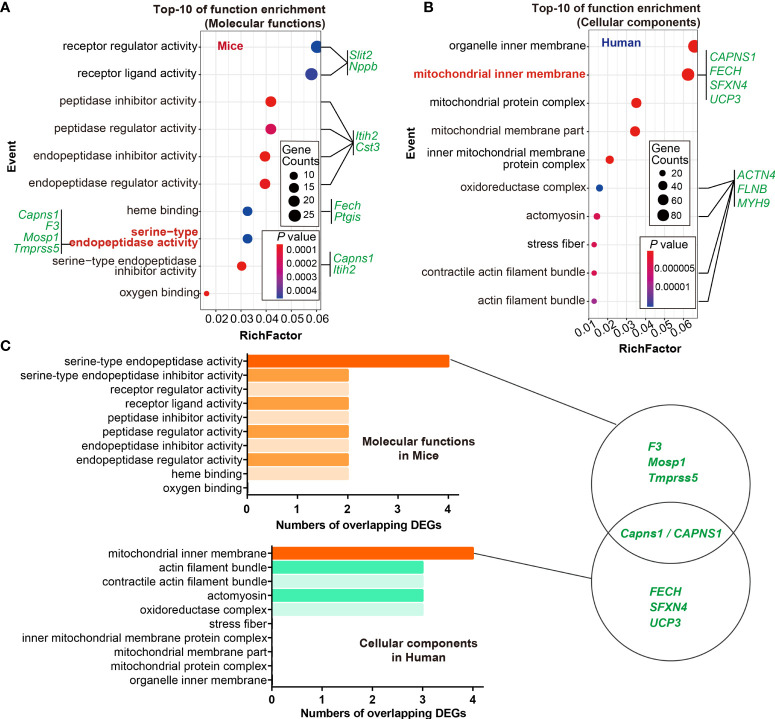
GO functional enrichment and module analysis of DEGs in the three datasets. **(A, B)** Bubble maps showed the top-10 GO events associated with DEGs of molecular functions module and cellular components module. Significantly enriched functions of two modules were indicated in *Y*-axis. Rich factor in the *X*-axis represented the enrichment levels. The larger value of Rich factor represented the higher level of enrichment. Color of the dot stands for the different *P*-value and size of the dot reflected the number of target genes enriched in the corresponding functions. Green letters in both modules indicated the overlapping DEGs. **(C)** Frequency of crucial overlapping DEGs (≥ 2 times) associated with top-10 events in both modules through GO analysis. There are seven crucial genes in T2D hearts according to the gene abundance grades in single event among the top-10 GO events, including *Capns1, F*3, *Mosp1*, *Tmpress5* in cellular components module, and *CAPNS1*, *FECH*, *SFXN4*, *UCP3* in molecular function module. *P* < 0.05. Schematics of GO analysis of overlapping DEGs for datasets GSE150316, GSE133054, and GSE161931 from NCBI-GEO (left panel). The richest DEGs enriching in corresponding functions showed in circles, and *CAPNS1* was the unique crucial gene shared by both modules (right panel).

**Figure 3:**
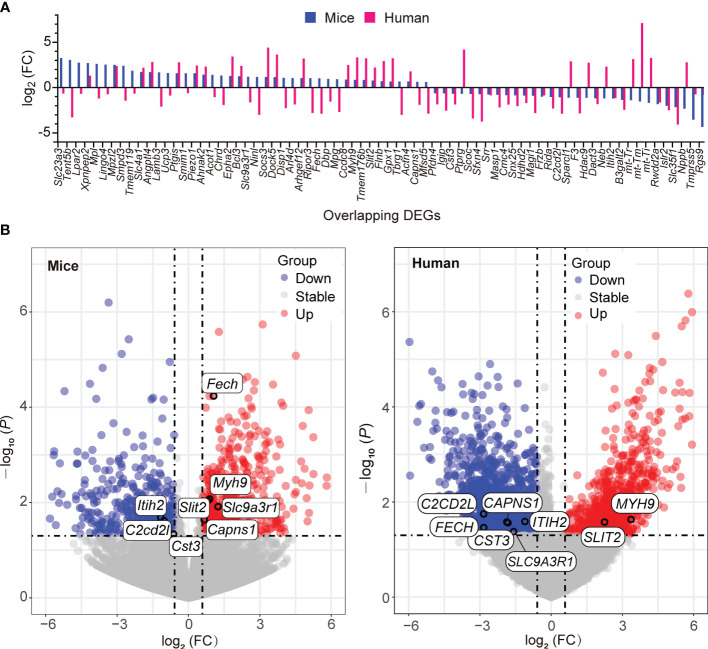
Transcription of the overlapping homologous DEGs with significance in T2D hearts. **(A)** Spectrum of the up- and down-regulation levels of the overlapping homologous DEGs in the datasets GSE161931 (mice), GSE150316 *vs* GSE133054 (human). **(B)** Distributions of the up- and down-regulated overlapping DEGs among the top-10 GO events in respective volcano plots.

Given that gene ontology enrichment is significant to elucidate the mechanisms of DEGs, we further performed GO function and KEGG pathway enrichment analyses to investigate the biological functions of DEGs from the three datasets through clusterProfiler package. The DEGs were examined and based on the *P*- value, the top 10 best ranked gene annotations were considered for further analysis for three sub-ontologies as follows, biological process, molecular functions and cellular components (30). The functions of DEGs were mainly enriched and described by dot plots as the top-10 GO events (**Figures 2A, B** and **Supplementary Figures 1A-C**). As could be seen, several dot plots showed overlapping homologous DEGs. The frequency of the top-10 GO events containing overlapping homologous DEGs was subsequently explored. Both serine-type endopeptidase activity in molecular function module and mitochondrial inner membrane in cellular components module contained more overlapping homologous DEGs than the others in the top-10 GO events (**Figures 2A, B**). There are seven crucial genes in T2D hearts according to the gene abundance grades in single event among the top-10 GO events, including *Capns1*, *F3*, *Mosp1*, *Tmpress5* in molecular function module and *CAPNS1*, *FECH*, *SFXN4*, *UCP3* in cellular components module. KEGG analyses were also presented in ridge plot format (**Supplementary Figure 1D**), both dot plot and ridge plot displayed the significantly altered events in T2D hearts. In the case of SARS-CoV-2 infection, mitochondria exhibited the most significant ranked gene annotations in cellular components of GO function enrichment in T2D hearts. Following the findings, we further observed that a unique gene *CAPNS1*/*Capna1* (calpain small subunit 1) was significantly enriched in serine-type endopeptidase of molecular function module and mitochondrial inner membrane of cellular components module in T2D hearts (**Figure 2C**). Owing to setting the cut-off criterion as genes with FC > 1.5 (or <1/1.5) and *P* < 0.05 to identified DEGs between T2D hearts and control samples, parts of DEGs included positive or negative regulation in transcription for the same gene in the heart samples of T2D (**Figure 3A**). The transcription level of *CAPNS1*/*Capna1* in hearts significantly increased in T2D mice, but surprisingly decreased in T2D patients who succumbed to SARS-CoV-2 infection (**Figures 3A, B**). The results suggested that SARS-CoV-2 infection did cause the transcriptional changes of gene in T2D hearts.

### PPI Network Construction and Module Analysis

The DEG-encoded proteins and their interactions among each other can provide a valuable clue (31). The PPI network was constructed with DEGs from all the three public datasets of NCBI-GEO described above (**Supplementary Figures 2A, B**). PPI analysis revealed there were 30 pairs of interactions in the overlapping homologous DEGs (**Supplementary Figure 2C**). These proteins were selected based on a combined score ≥ 0.7 in STRING analysis. Consistently, molecule CAPNS1 shared by molecular function module and cellular components module in transcription were also displayed in the interaction of PPI network (**Supplementary Figure 2C**). The results supported that the most significant function of DEGs were also enriched in their encoded proteins and their interactions to each other in T2D hearts, respectively.

### Enhanced Cardiac *Capns1* Transcription Association With JP2 Proteolysis in *db/db* Mice

**Figure 4:**
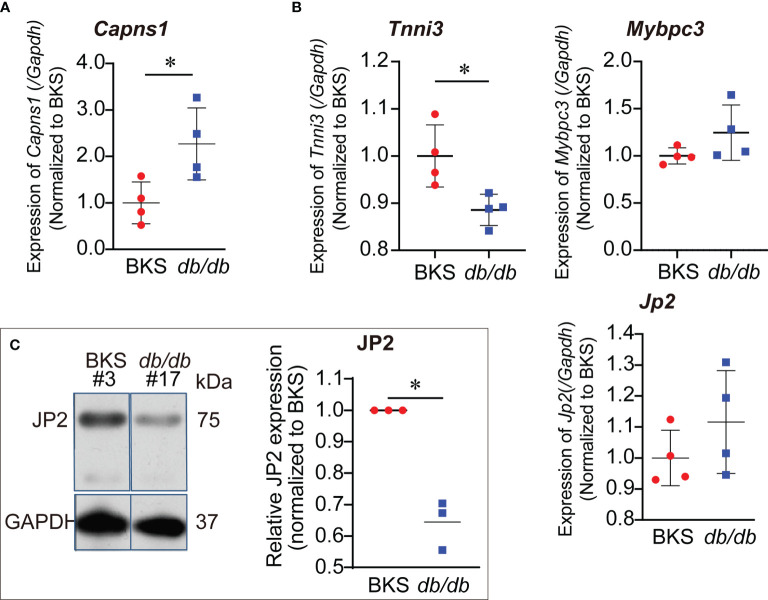
Increased transcription of *Capns1* in the hearts of *db/db* mice. **(A)** Cardiac *Capns1* transcription enhanced in *db/db* mice (16-weeks-old). **(B)** The transcription levels of substrates *Jp2*, *Tnni3*, and *Mybpc3* in cardiac calpain/CAPNS1 pathway were not reduced (*n*=4, each sample was repeated three times). **(C)** Representative blotting showing JP2 protein decreased in expression in T2D hearts (*n*=3, each sample was repeated three times, #3 and #17 were original lab codes of animals). *P < 0.05.

Calpain proteolysis contributes to the pathogenesis of heart failure. CAPNS1 was a regulatory subunit of calpain, both functional enrichment of DEGs and PPI analysis revealed that calpain/CAPNS1 pathway might serve as a crucial target in T2D hearts. To explore whether proteolysis catalyzed by calpain/CAPNS1 was involved in the pathological process of T2D hearts, we first observed cardiac *Capns1* mRNA level in *db/db* mice at the age of 16 weeks (late stage of T2D heart progress). As showed in **Figure 4A**, *Capns1* transcription significantly enhanced in the hearts of *db/db* mice, reached 2.29 fold of that in control. We then examined the transcription levels of the substrates, *Jp2*, *Tnni3*, and *Mybpc3*, catalyzed by calpain/CAPNS1 in the hearts of *db/db* mice. Compared to that in control, *Jp2* and *Mybpc3* did not change in transcription, while *Tnni3* showed significant decrease (**Figure 4B**). It suggested that the transcription of substrates *Jp2* and *Mybpc3* to calpain/CAPNS1was stable. The attenuation of troponin 1 type 3 (*Tnni3*) in T2D heart appeared as early as in transcriptional step in a non-enzymatic catalytic manner. Furthermore, the relative expression of JP2 declined to 32.2 ± 3.9% compared to control by Western blot assay (**Figure 4C**). These results indicated that substrate JP2 of calpain/CAPNS1 did undergo a proteolysis process.

### Abnormalities of Mitochondrial Ultrastructure in *db/db* Mice

**Figure 5:**
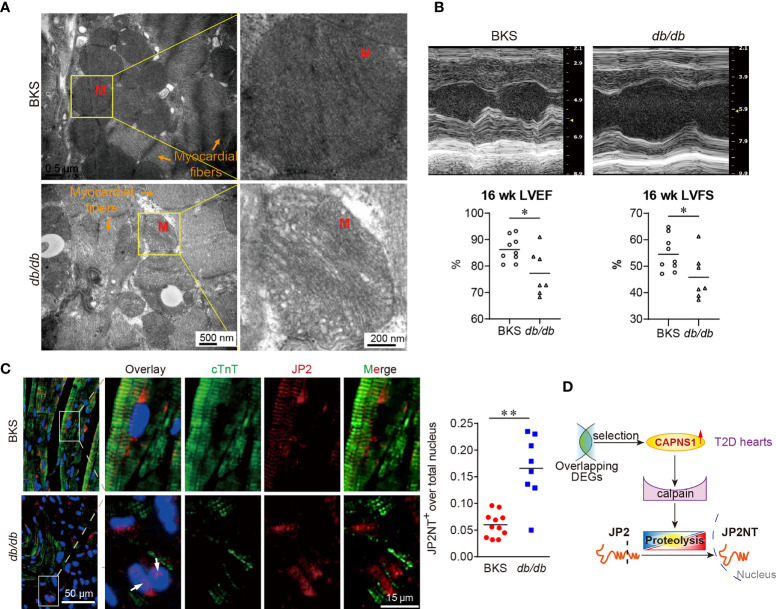
Mitochondrial cristae disruption and hydrolyzed JP2 nuclear translocation in myocardium of *db/db* mice. **(A)** Electron micrographs showed the decreased areas of cristae organization and density of mitochondria (M) in *db/db* mice compared with controls. Scale bar: 500 nm and 200 nm. **(B)** Representative images of echocardiography. Both left ventricular ejection fraction (LVEF) and left ventricular fraction shortening (LVFS) decreased. *P < 0.05, *n*=9 (BKS mice), *n*=7 (*db/db* mice). *P < 0.05, **P < 0.01. **(C)** The abundance and distribution of JP2 (red) changed in cardiomyocytes (green, cTnT) of T2D heart. The colocalization of JP2NT (red) with nuclei (blue, DAPI) appear in cardiomyocytes of T2D heart (arrow: nuclear JP2NT). **(D)** In the case of cardiac mitochondrial ultrastructure lesions, and cardiac dysfunction in T2D hearts, the hydrolyzed product JP2NT import into the nucleus of myocardium. Scale bar: 50 μm and 15 μm (left panel). Ratio of JP2NT existed in nuclei were showed in dot chart (right panel) (n=8~11, 4 random fields for each sample).

Considering that in the case of SARS-CoV-2 infection, mitochondria exhibited the most significant ranked gene annotations in cellular components of GO function enrichment in T2D hearts, we further examined cardiac mitochondrial ultrastructure in *db/db* mice at the age of 16 weeks by transmission electron microscopy. Qualitative analysis of electron micrographs showed disorganized mitochondrial cristae (inner membrane) and diminished cristae density in the hearts of *db/db* mice in comparison with those in controls (**Figure 5A**). Meanwhile, echocardiographic analysis demonstrated left ventricular dysfunction in *db/db* mice. Compared to control, both left ventricular ejection fraction (LVEF) and left ventricular fraction shortening (LVFS) were significantly decreased in the T2D hearts (**Figure 5B**). Taken together, in the case of left ventricular dysfunction in T2D progress, *CAPNS1* located in the worse cardiac mitochondria cristae might enhance in transcription, thereby contributing to trigger substrate JP2 proteolysis.

### JP2NT Nucleus Translocation in the Hearts of *db/db* Mice

To explore whether or not substrate JP2 proteolysis took a role in T2D hearts, we used immunofluorescence confocal image to analyses the myocardium of *db/db* mice. We focused on the abundance and distribution of JP2 in cardiomyocytes of T2D heart. The sarcomere structure of cardiac troponin T (cTnT) seriously damaged and dramatically reduced. Compared to that in control, the distribution of JP2 displayed completely separating from cTnT in T2D hearts (**Figure 5C**, left panel). On the other hand, hydrolyzed N-terminal fragment of JP2 (JP2-NT) imported into the nuclei of myocardium in *db/db* mice, which characterized by the overlay of JP2-NT with DAPI in cTnT positive cells (**Figure 5C**, arrows in left panel). The ratio of JP2-NT localized in nuclei was significantly increased in T2D hearts compared with control (**Figure 5C**, right panel). In the present study, we revealed a novel regulating mechanism in the late stage of T2D heart progress. *CAPNS1* increased in transcription at the mitochondria cristae, which favored the substrate JP2 to calpain/CAPNS1 hydrolysis and nuclear translocation.

## Discussion

The available data have supported that increased susceptibility in patients with T2D to SARS-CoV-2 hospitalizations (5, 6). Moreover, an acute cardiovascular manifestation of COVID-19 often presents as an acute cardiac injury in the absence of obstructive coronary artery disease (7, 8). SARS-CoV-2 directly infects cardiomyocytes *in vitro* or in T2D patients in an ACE2-dependent manner (6, 11). But the underlying mechanism of how SARS-CoV-2 damages the heart remains to be elucidated. The present study attempts to identify genomic differences between T2D- and non-T2D hearts and also signify transcriptomic effects of SARS-CoV-2 infection on the hearts through a number of bioinformatics approaches.

The primary principle in R package is more suitable for our datasets. Since the R package provides a straight forward method for visualizing DGE result files that from the most commonly used DGE tools: DESeq2, edger and Cuffdiff. Nine functions are provided, including six distinct visualizations with three matrix options. In R package, the regularly updated tools can provide continuous support in the long run. DESeq2 is designed for differential gene expression analysis of RNA-seq data, especially for those experiments with small numbers of replicates, and allow a more general, data-driven parameter estimation (22).

In the current study, we perform bioinformatics analysis on the expression profiles of public datasets and identify a pathogenic and prognostic gene *CAPNS1* in T2D hearts. We reveal that T2D hearts themselves originally over-transcribe *CAPNS1*, a gene of regulatory subunit of calpain. Using bioinformatics analysis including GO function enrichment analysis, KEGG pathway analysis, and PPI network analysis, we find that serine-type endopeptidase activity in molecular function module, and mitochondria inner membrane in cellular component module are the major GO enriched events for the overlapping homologous DEGs. The upregulated and downregulated genes suggest that the expression and/or transcription of genes in T2D hearts undergo robust changes under the SARS-CoV-2 infection condition. In the present study, there are seven crucial genes in T2D hearts according to the gene abundance grades in single event among the top-10 GO events, including *Capns1, F*3, *Mosp1*, *Tmpress5* in cellular component module, and *CAPNS1*, *FECH*, *SFXN4*, *UCP3* in molecular function module. From the cellular components point of view, most crucial genes locate in mitochondria. The most valuable finding is that *CAPNS1* is the unique crucial gene shared by both molecular function module and cellular components module of the overlapping DEGs in T2D hearts. It means that transcriptional level of *CAPNS1* is extremely crucial for the T2D hearts. Transcriptional regulation is generally considered the mode of choice to adapt to chronic stimuli or diseases (6), markedly increase of *CAPNS1* mRNA does indicate its meaningful significance to adapt to the pathological function of T2D hearts. We therefore focus on the cardiac calpain/CAPNS1 pathway, and subsequently observe its biological function in *db/db* mice.

Calpain/CAPNS1 together form active μ-calpain and possess a mitochondrial targeting sequence in the N-terminal region of calpain (32). Calpain/CAPNS1 activation catalyzes substrates proteolysis. The present study has confirmed that cardiac *Capns1* increase in transcription in *db/db* T2D mice. As a regulate subunit, over-transcribed *CAPNS1* holds the more possibility to subsequently together with calpain, and favors μ-calpain formation in the mitochondria inner membrane of T2D hearts. JP2, Tnni3, and Mybpc3 are the substrates catalyzed by Calpain/CAPNS1 in the hearts. Mutations of *Jp2*, *Tnni3*, and *Mybpc3* are closely relevant to the heart diseases. Our results demonstrate that the transcription of *Jp2* and *Mybpc3* was stable in T2D hearts. The attenuation of *Tnni3* in T2D heart appeared as early as in transcriptional step in a non-enzymatic catalytic manner. The results suggest less effects of calpain/CAPNS1 on the transcription of its substrates. On the other hands, JP2 protein significantly decreases and its distribution exhibits separation from cTnT in cardiomyocytes of *db/db* mice, indicating that JP2 undergoes both quantitatively and functionally changes in cytoplasm. Hydrolyzed N-terminal fragment of JP2 (JP2NT) can trigger a pathway in the heart pathophysiology (33, 34). By this pathway, a self-protective mechanism that enables failing cardiomyocytes in the stressed myocardium to transduce mechanical information into salutary transcription reprogramming. But so far the upstream mechanism of this pathway is not elucidated. Current study further confirms that in the case of cardiac mitochondrial ultrastructure lesions, and cardiac dysfunction in *db/db* T2D mice, the hydrolyzed product JP2NT import into the nucleus of myocardium. Considering that *CAPNS1* is the unique gene not only shared by molecular function module and cellular component module of overlapped DEGs, but also involved in catalyzing JP2 hydrolysis, we suggest that T2D hearts should possess intrinsic CAPNS1-dependent self-protective mechanism. Based on our knowledge, this is the first time to reveal that cardiac *CAPNS1* overtranscription associated with JP2 hydrolysis might serve as a switch to initiate a compensatory role in the T2D hearts.

**Figure 6:**
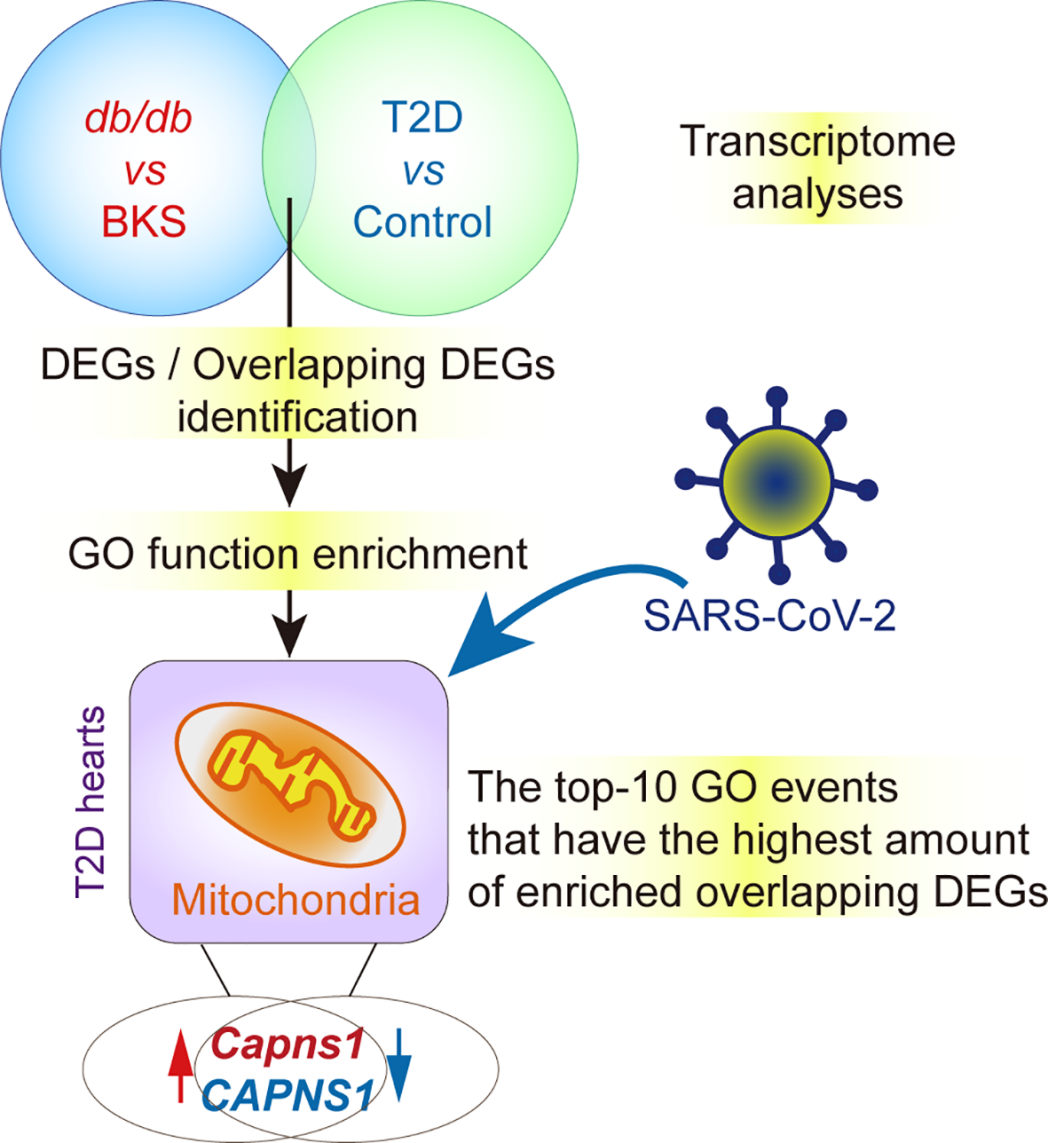
∣ Hypothesis contributing to crucial gene and key functions in T2D hearts with and without SARS-CoV-2 infection. *Capns1*/*CAPNS1* is the unique gene shared by molecular function module and cellular components module of overlapped DEGs, and involved in catalyzing JP2 hydrolysis, T2D hearts should possess intrinsic CAPNS1-dependent self-protective mechanism. The autopsy heart specimens of T2D patients display attenuated transcription of *CAPNS1* in the case of SARS-CoV-2 infection. Attenuated cardiac *CAPNS1* transcription in T2D patients who succumbed to SARS-CoV-2 infection must decrease in the ability to hydrolyze JP2 and weaken self-protective mechanism, thereby leading to adverse prognostics.

Given that both animal and human with diabetic cardiomyopathy share the same potential biomarkers (19), applying bioinformatic analysis to identify the potential pathogenic and prognostic DEGs are extremely valuable for the T2D heart samples of *db/db* mice and patients. The present study demonstrates the autopsy heart specimens of T2D patients display attenuated transcription of *CAPNS1* in the case of SARS-CoV-2 infection. Based on the findings, we suggested that attenuated cardiac *CAPNS1* transcription in T2D patients who succumbed to SARS-CoV-2 infection must decrease in the ability to hydrolyze JP2 and weaken self-protective mechanism, thereby leading to adverse prognostics (**Figure 6**). This finding is currently not included in the mechanisms for adverse outcomes of COVID-19. It surely takes an important contribution to deeply consummate the statement that SARS-CoV-2 directly infects cardiomyocytes *in vitro* and in T2D patients in an ACE2-dependent manner (6, 11). Based on our findings, acute cardiac injury should be one of the independent leading causes of adverse outcomes and death in T2D patients after SARS-CoV-2 infection, although COVID-19 often causes clotting issues in the coronaries of those sick. *Db/db* mice at 16-weeks-old are in the late stages of T2D progress. The current study identifies the overlapping homologous DEGs and conducts a series of bioinformatics analysis to screen the unique gene and pathway at a genome-wide scale through analyzing T2D datasets. SARS-CoV-2 infects vascular endothelium can trigger mitochondrial reactive oxygen species production and glycolytic shift (35, 36). S protein alone can damage vascular endothelial cells by down-regulating ACE2 and consequently inhibiting mitochondrial function (37). Mitochondrial dysfunction suggests that deleterious changes in mitochondria occurring in the heart in the context of T2D (38). Though mitochondrial redox state changes occur in the heart with obesity and diabetes, how it connects the remodeled energy metabolism with mitochondrial and cytosolic antioxidant defense and nuclear epigenetic changes remains to be determined (39). The underlying mechanism of SARS-CoV-2 infects cardiomyocytes mainly involves endoplasmic reticulum stress combined with mitochondria dysfunction and apoptosis (6, 11). Our findings in decreased transcription of *CAPNS1* in mitochondria inner membrane after SARS-CoV-2 infects and mitochondrial ultrastructure damage further support the putative mechanism. It may be attributable to poor mitochondrial condition triggering *CAPNS1* overexpression. In the case of SARS-CoV-2 infection, diminishing calpain/CAPNS1-associated self-protective mechanism could lead to T2D heart decompensatory.

This evidence implies a possibility that SARS-CoV-2 infection must diminish the self-protective mechanism by inhibiting *CAPNS1* transcription in the hearts of T2D patients. Based on described above, we suggest that lost cardiac *CAPNS1* gene should disturb JP2NT regulated salutary transcription reprogramming in the T2D severe cases with COVID-19. These results collectively suggest that a comprehensive investigation of these overlapping DEGs will facilitate our understanding of acute cardiac injury in T2D patients after SARS-CoV-2 infection. Therefore, *CAPNS1* gene may serve as a potential biomarker and therapeutic target for the onset, progression and prognostic of cardiovascular syndrome in T2D patients with SARS-CoV-2 infection, and a potential target of anti-SARS-CoV-2 comprehensive therapy, although it remains to be validated by further pre-clinical and prospective clinical studies (40).

Our transcriptomics based approach using T2D heart tissues of mouse and human (heart specimens with COVID-19) as a novel candidate progressive, prognostic and pharmacological target associated with T2D patients’ anti-SARS-CoV-2 comprehensive therapy. However, there are several shortcomings of this study with regards to the role of CAPSN1 in the context of T2D hearts. First of all, we do not consider applying bottleneck method to explore hub genes, in this way analysis of the total amount of information probably will become easier. Otherwise we might be better predict the target and get a better representation. Next, given the fact that the heart specimens in T2D patients with COVID-19 are quite heterogeneous, further experiments and efforts to identify *Capns1* in mice mimicking SARS-CoV-2 infection appear well justified. The impact of inhibition of cardiac CAPSN1 protein function on T2D mice might also be needed. Although limitations of the current study, these are multiple research avenues to be further explored, for which the presented study highlights a novel starting point.

## Data Availability Statement

The datasets presented in this study can be found in online repositories. The names of the repository/repositories and accession number(s) can be found in the article/**Supplementary Material**.

## Ethics Statement

The animal study was reviewed and approved by Animal Care and Ethics Committee of Zhejiang University, and 2nd Affiliated Hospital Research Ethics Committee of Zhejiang University, China.

## Author Contributions

XH conceived, designed the research, conducted the bioinformatics and molecular biological experiments, provided part of the financial and instrumental support, analyzed the data and wrote the manuscript. K-jZ and J-jJ, participated in the major collection of animal experiments, electron micrograph and analysis. S-yJ and J-bL performed the echocardiographic collection and analysis. Y-jL conducted part of the animal experimental design and provided part of the financial and instrumental support. All authors read and approved the final manuscript.

## Funding

This work was supported by the National Natural Science Foundation of China (NSFC, No. 81700445 to XH, and No.81573513 to Y-jL); also by The opening foundation of the State Key Laboratory for Diagnosis and Treatment of Infectious Diseases and Collaborative Innovation Center for Diagnosis and Treatment of Infectious Diseases, The First Affiliated Hospital, College of Medicine, Zhejiang University (grant NO. SKLID2019KF05 to XH).

## Conflict of Interest

The authors declare that the research was conducted in the absence of any commercial or financial relationships that could be construed as a potential conflict of interest.

## Publisher’s Note

All claims expressed in this article are solely those of the authors and do not necessarily represent those of their affiliated organizations, or those of the publisher, the editors and the reviewers. Any product that may be evaluated in this article, or claim that may be made by its manufacturer, is not guaranteed or endorsed by the publisher.

## Supplementary Material

Supplementary Figure 1 ∣ GO and KEGG enrichment analysis of overlapping DEGs. **(A, B)** Bubble maps showed the top-10 GO events associated with overlapping DEGs (green color). *P* < 0.05. The larger value of gene ratio represents the higher level of enrichment. The color of the dot stands for the different *P*-value, and the size of the dot reflects the number of target genes enriched in the corresponding event. **(C)** Volcano plots of sifted out DEGs for dataset GSE150316 *vs* GSE133054 (Human), and GSE161931 (Mice). **(D)** Ridge plots of KEGG analysis for DEGs. *P* < 0.05.

Supplementary Figure 2 ∣ Protein-protein interaction (PPI) network construction. **(A, B)** PPI network constructed with the DEGs from all the three public datasets of GEO. **(C)** The significant module identified from the PPI network of overlapping homologous DEGs using the molecular complex detection (MCODE) method with a score of ≥ 5.0.

## References

[1] Richardson S Hirsch JS Narasimhan M Crawford JM McGinn T Davidson KW. Presenting Characteristics, Comorbidities, and Outcomes Among 5700 Patients Hospitalized With COVID-19 in the New York City Area. JAMA (2020) 323(20):2052–9. doi: 10.1001/jama.2020.6775 PMC7177629 32320003

[2] Bloomgarden ZT. Diabetes and COVID-19. J Diabetes (2020) 12(4):347–8. doi: 10.1111/1753-0407.13027 32162476

[3] Muniyappa R Gubbi S. COVID-19 Pandemic, Coronaviruses, and Diabetes Mellitus. Am J Physiol Endocrinol Metab (2020) 318(5):E736–E41. doi: 10.1152/ajpendo.00124.2020 PMC7191633 32228322

[4] Sardu C Gargiulo G Esposito G Paolisso G Marfella R. Impact of Diabetes Mellitus on Clinical Outcomes in Patients Affected by Covid-19. Cardiovasc Diabetol (2020) 19(1):76. doi: 10.1186/s12933-020-01047-y 32527257 PMC7289072

[5] Drucker DJ. Coronavirus Infections and Type 2 Diabetes-Shared Pathways With Therapeutic Implications. Endocr Rev (2020) 41(3):1–13. doi: 10.1210/endrev/bnaa011 PMC7184382 32294179

[6] D’Onofrio N Scisciola L Sardu C Trotta MC De Feo M Maiello C. Glycated ACE2 Receptor in Diabetes: Open Door for SARS-COV-2 Entry in Cardiomyocyte. Cardiovasc Diabetol (2021) 20(1):99. doi: 10.1186/s12933-021-01286-7 33962629 PMC8104461

[7] Hendren NS Drazner MH Bozkurt B Cooper LT Jr. Description and Proposed Management of the Acute COVID-19 Cardiovascular Syndrome. Circulation (2020) 141(23):1903–14. doi: 10.1161/CIRCULATIONAHA.120.047349 PMC7314493 32297796

[8] Fried JA Ramasubbu K Bhatt R Topkara VK Clerkin KJ Horn E. The Variety of Cardiovascular Presentations of COVID-19. Circulation (2020) 141(23):1930–6. doi: 10.1161/CIRCULATIONAHA.120.047164 PMC7314498 32243205

[9] Angelidi AM Belanger MJ Mantzoros CS. Commentary: COVID-19 and Diabetes Mellitus: What We Know, How Our Patients Should be Treated Now, and What Should Happen Next. Metabolism (2020) 107:154245. doi: 10.1016/j.metabol.2020.154245 32320742 PMC7167295

[10] Rubino F Amiel SA Zimmet P Alberti G Bornstein S Eckel RH. New-Onset Diabetes in Covid-19. N Engl J Med (2020) 383(8):789–90. doi: 10.1056/NEJMc2018688 PMC7304415 32530585

[11] Bojkova D Wagner JUG Shumliakivska M Aslan GS Saleem U Hansen A. SARS-CoV-2 Infects and Induces Cytotoxic Effects in Human Cardiomyocytes. Cardiovasc Res (2020) 116(14):2207–15. doi: 10.1093/cvr/cvaa267 PMC7543363 32966582

[12] Sukumaran V Tsuchimochi H Tatsumi E Shirai M Pearson JT. Azilsartan Ameliorates Diabetic Cardiomyopathy in Young Db/Db Mice Through the Modulation of ACE-2/ANG 1-7/Mas Receptor Cascade. Biochem Pharmacol (2017) 144:90–9. doi: 10.1016/j.bcp.2017.07.022 28789938

[13] Ji AL Rubin AJ Thrane K Jiang S Reynolds DL Meyers RM. Multimodal Analysis of Composition and Spatial Architecture in Human Squamous Cell Carcinoma. Cell (2020) 182(6):1661–2. doi: 10.1016/j.cell.2020.08.043 PMC7505493 32946785

[14] Deng JL Xu YH Wang G. Identification of Potential Crucial Genes and Key Pathways in Breast Cancer Using Bioinformatic Analysis. Front Genet (2019) 10:695. doi: 10.3389/fgene.2019.00695 31428132 PMC6688090

[15] Nashiry A Sarmin Sumi S Islam S Quinn JMW Moni MA. Bioinformatics and System Biology Approach to Identify the Influences of COVID-19 on Cardiovascular and Hypertensive Comorbidities. Brief Bioinform (2021) 22(2):1387–401. doi: 10.1093/bib/bbaa426 PMC7929376 33458761

[16] Taz TA Ahmed K Paul BK Kawsar M Aktar N Mahmud SMH. Network-Based Identification Genetic Effect of SARS-CoV-2 Infections to Idiopathic Pulmonary Fibrosis (IPF) Patients. Brief Bioinform (2020) 22(2):1254–66. doi: 10.1093/bib/bbaa235 PMC7665362 33024988

[17] Taz TA Ahmed K Paul BK Al-Zahrani FA Mahmud SMH Moni MA. Identification of Biomarkers and Pathways for the SARS-CoV-2 Infections That Make Complexities in Pulmonary Arterial Hypertension Patients. Brief Bioinform (2021) 22(2):1451–65. doi: 10.1093/bib/bbab026 PMC7929374 33611340

[18] Desai N Neyaz A Szabolcs A Shih AR Chen JH Thapar V. Temporal and Spatial Heterogeneity of Host Response to SARS-CoV-2 Pulmonary Infection. Nat Commun (2020) 11(1):6319. doi: 10.1038/s41467-020-20139-7 33298930 PMC7725958

[19] Lee MMY McMurray JJV Lorenzo-Almoros A Kristensen SL Sattar N Jhund PS. Diabetic Cardiomyopathy. Heart (2019) 105(4):337–45. doi: 10.1136/heartjnl-2016-310342 30337334

[20] Edgar R Domrachev M Lash AE. Gene Expression Omnibus: NCBI Gene Expression and Hybridization Array Data Repository. Nucleic Acids Res (2002) 30(1):207–10. doi: 10.1093/nar/30.1.207 PMC99122 11752295

[21] Brazma A. Minimum Information About a Microarray Experiment (MIAME)–Successes, Failures, Challenges. ScientificWorldJournal (2009) 9:420–3. doi: 10.1100/tsw.2009.57 PMC5823224 19484163

[22] McDermaid A Monier B Zhao J Liu B Ma Q. Interpretation of Differential Gene Expression Results of RNA-Seq Data: Review and Integration. Brief Bioinform (2019) 20(6):2044–54. doi: 10.1093/bib/bby067 PMC6954399 30099484

[23] Mahmud SMH Al-Mustanjid M Akter F Rahman MS Ahmed K Rahman MH. Bioinformatics and System Biology Approach to Identify the Influences of SARS-CoV-2 Infections to Idiopathic Pulmonary Fibrosis and Chronic Obstructive Pulmonary Disease Patients. Brief Bioinform (2021) 22(5):1–20. doi: 10.1093/bib/bbab115 33847347 PMC8083324

[24] Mokou M Klein J Makridakis M Bitsika V Bascands JL Saulnier-Blache JS. Proteomics Based Identification of KDM5 Histone Demethylases Associated With Cardiovascular Disease. EBioMedicine (2019) 41:91–104. doi: 10.1016/j.ebiom.2019.02.040 30826357 PMC6443027

[25] Gene Ontology C. The Gene Ontology Resource: Enriching a GOld Mine. Nucleic Acids Res (2021) 49(D1):D325–D34. doi: 10.1093/nar/gkaa1113 PMC7779012 33290552

[26] Bader GD Hogue CW. An Automated Method for Finding Molecular Complexes in Large Protein Interaction Networks. BMC Bioinf (2003) 4:2. doi: 10.1186/1471-2105-4-2 PMC149346 12525261

[27] Szklarczyk D Gable AL Nastou KC Lyon D Kirsch R Pyysalo S. The STRING Database in 2021: Customizable Protein-Protein Networks, and Functional Characterization of User-Uploaded Gene/Measurement Sets. Nucleic Acids Res (2021) 49(D1):D605–D12. doi: 10.1093/nar/gkaa1074 PMC7779004 33237311

[28] Coelho-Filho OR Shah RV Mitchell R Neilan TG Moreno H Jr. Simonson B. Quantification of Cardiomyocyte Hypertrophy by Cardiac Magnetic Resonance: Implications for Early Cardiac Remodeling. Circulation (2013) 128(11):1225–33. doi: 10.1161/CIRCULATIONAHA.112.000438 PMC5308548 23912910

[29] Li L Li T Zhang Y Pan Z Wu B Huang X. Peroxisome Proliferator-Activated Receptorbeta/Delta Activation is Essential for Modulating P-Foxo1/Foxo1 Status in Functional Insulin-Positive Cell Differentiation. Cell Death Dis (2015) 6:e1715. doi: 10.1038/cddis.2015.88 25855963 PMC4650555

[30] Sun Y Yuan K Zhang P Ma R Zhang QW Tian XS. Crosstalk Analysis of Pathways in Breast Cancer Using a Network Model Based on Overlapping Differentially Expressed Genes. Exp Ther Med (2015) 10(2):743–8. doi: 10.3892/etm.2015.2527 PMC4509018 26622386

[31] Szklarczyk D Morris JH Cook H Kuhn M Wyder S Simonovic M. The STRING Database in 2017: Quality-Controlled Protein-Protein Association Networks, Made Broadly Accessible. Nucleic Acids Res (2017) 45(D1):D362–D8. doi: 10.1093/nar/gkw937 PMC5210637 27924014

[32] Badugu R Garcia M Bondada V Joshi A Geddes JW. N Terminus of Calpain 1 Is a Mitochondrial Targeting Sequence. J Biol Chem (2008) 283(6):3409–17. doi: 10.1074/jbc.M706851200 18070881

[33] Lim GB. Protective Transcriptional Repression by Junctophilin 2 Fragment. Nat Rev Cardiol (2019) 16(1):5. doi: 10.1038/s41569-018-0130-9 30487546

[34] Guo A Wang Y Chen B Yuan J Zhang L Hall D. E-C Coupling Structural Protein Junctophilin-2 Encodes a Stress-Adaptive Transcription Regulator. Science (2018) 362(6421):1–9. doi: 10.1126/science.aan3303 PMC6336677 30409805

[35] Teuwen LA Geldhof V Pasut A Carmeliet P. COVID-19: The Vasculature Unleashed. Nat Rev Immunol (2020) 20(7):389–91. doi: 10.1038/s41577-020-0343-0 PMC7240244 32439870

[36] Codo AC Davanzo GG Monteiro LB de Souza GF Muraro SP Virgilio-da-Silva JV. Elevated Glucose Levels Favor SARS-CoV-2 Infection and Monocyte Response Through a HIF-1alpha/Glycolysis-Dependent Axis. Cell Metab (2020) 32(3):437–46 e5. doi: 10.2139/ssrn.3606770 32697943 PMC7367032

[37] Lei Y Zhang J Schiavon CR He M Chen L Shen H. SARS-CoV-2 Spike Protein Impairs Endothelial Function via Downregulation of ACE 2. Circ Res (2021) 128(9):1323–6. doi: 10.1161/CIRCRESAHA.121.318902 PMC8091897 33784827

[38] Gibb AA Epstein PN Uchida S Zheng Y McNally LA Obal D. Exercise-Induced Changes in Glucose Metabolism Promote Physiological Cardiac Growth. Circulation (2017) 136(22):2144–57. doi: 10.1161/CIRCULATIONAHA.117.028274 PMC5704654 28860122

[39] Berthiaume JM Kurdys JG Muntean DM Rosca MG. Mitochondrial NAD(+)/NADH Redox State and Diabetic Cardiomyopathy. Antioxid Redox Signal (2019) 30(3):375–98. doi: 10.1089/ars.2017.7415 PMC6306679 29073779

[40] Mahmud SMH Chen W Liu Y Awal MA Ahmed K Rahman MH. PreDTIs: Prediction of Drug-Target Interactions Based on Multiple Feature Information Using Gradient Boosting Framework With Data Balancing and Feature Selection Techniques. Brief Bioinform (2021) 22(5):1–20. doi: 10.1093/bib/bbab046 33709119 PMC7989622

