# Supplementary figures and images for: Identification of Crucial Genes and Key Functions in Type 2 Diabetic Hearts by Bioinformatic Analysis
Source: Front Endocrinol (Lausanne). 2022 Feb 15;13:801260. doi: 10.3389/fendo.2022.801260 (PMC8885996; doi:10.3389/fendo.2022.801260)

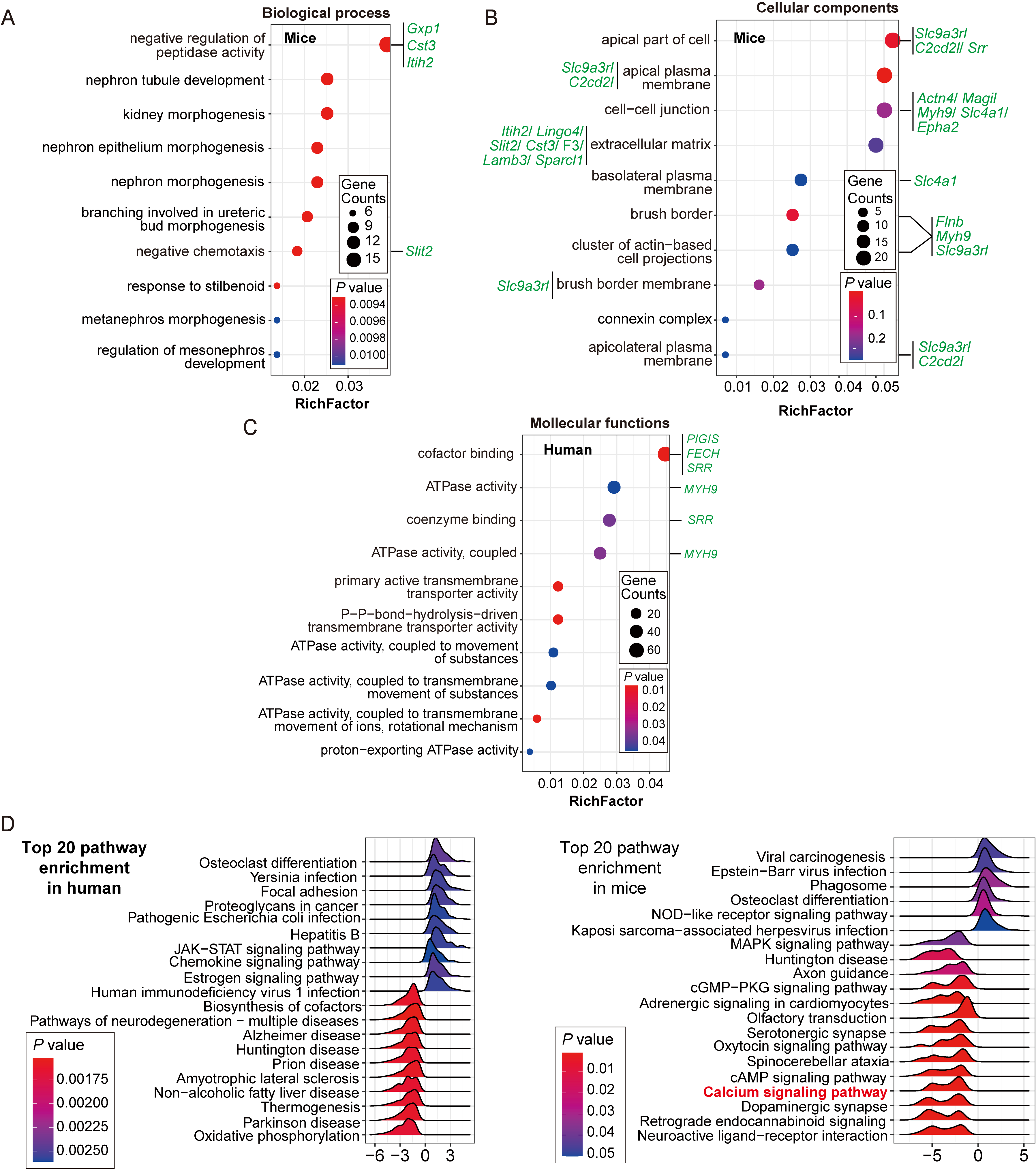

Supplement: Supplementary Figure 1 — ∣ GO and KEGG enrichment analysis of overlapping DEGs. (A, B) Bubble maps showed the top-10 GO events associated with overlapping DEGs (green color). P < 0.05. The larger value of gene ratio represents the higher level of enrichment. The color of the dot stands for the different P-value, and the size of the dot reflects the number of target genes enriched in the corresponding event. (C) Volcano plots of sifted out DEGs for dataset GSE150316 vs GSE133054 (Human), and GSE161931 (Mice). (D) Ridge plots of KEGG analysis for DEGs. P < 0.05. [file Image_1.tif]

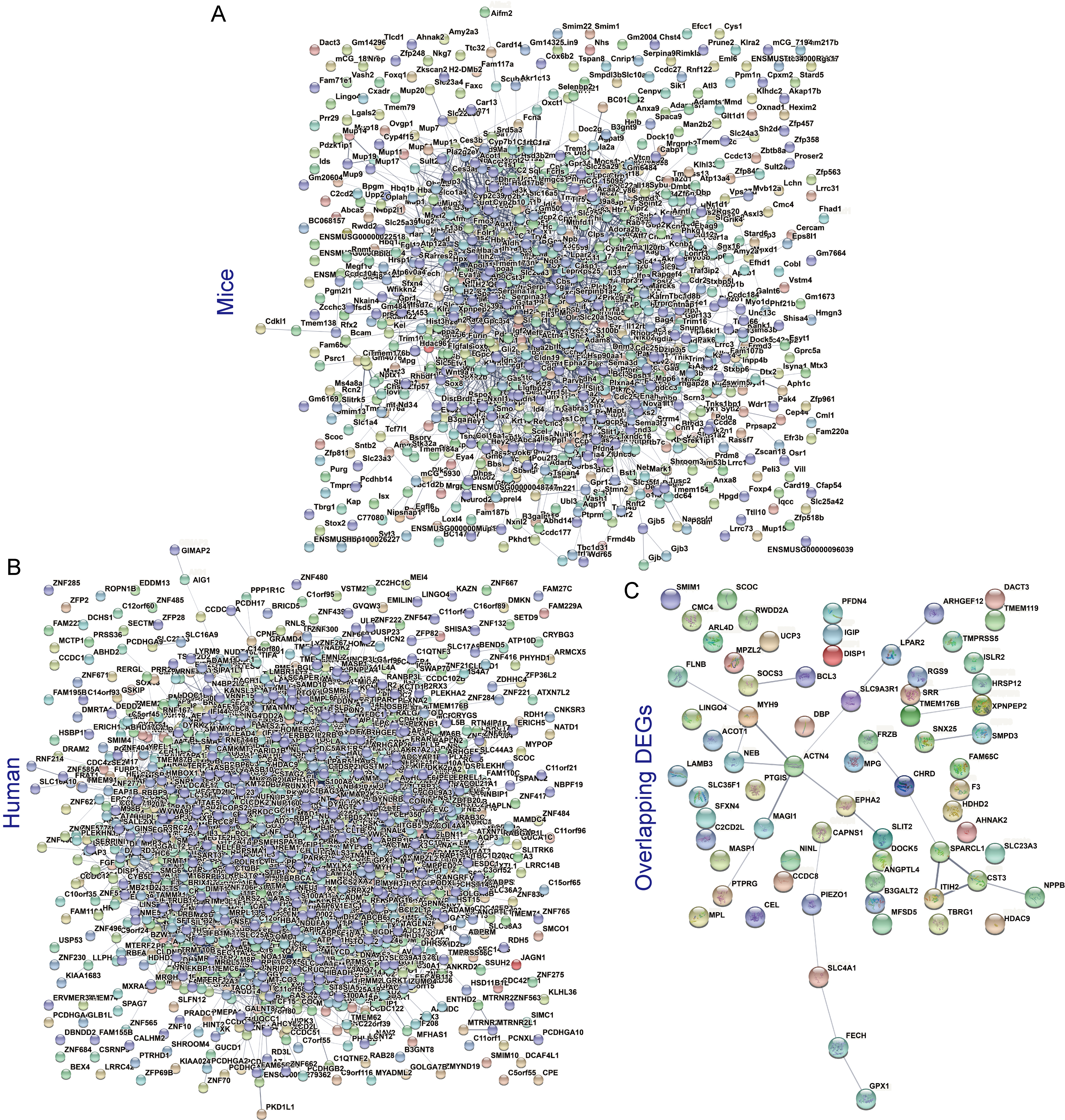

Supplement: Supplementary Figure 2 — ∣ Protein-protein interaction (PPI) network construction. (A, B) PPI network constructed with the DEGs from all the three public datasets of GEO. (C) The significant module identified from the PPI network of overlapping homologous DEGs using the molecular complex detection (MCODE) method with a score of ≥ 5.0. [file Image_2.tif]
