# Supplementary material for: Identification of Crucial Genes and Key Functions in Type 2 Diabetic Hearts by Bioinformatic Analysis
Source: Front Endocrinol (Lausanne). 2022 Feb 15;13:801260. doi: 10.3389/fendo.2022.801260 (PMC8885996; doi:10.3389/fendo.2022.801260)
Supplement: Supplementary file 3 [file Table_1.docx]

**TABLE S1. Name and sequence of primers sets for real-time RT-PCR**

| Gene name | Primer sequence |
| --- | --- |
| *Jp2* | Forward: CCAAGGGCCAGGGTGAATAC |
|  | Reverse: TGGCTCCAATATCCCTCAAAGG |
| *Tnni3* | Forward: TCTGCCAACTACCGAGCCTAT  Reverse: CTCTTCTGCCTCTCGTTCCAT |
| *Mybpc3* | Forward: CAGGGAAGAAACCAGTGTCAG  Reverse: GCTGCCAAACCATACTTGTCATT |
| *Capns1* | Forward: ATCCTGGGCGGAGTCATTAGT  Reverse: TTTCCTGAACTGACGGACCTC |
| *Gapdh* | Forward: GGTGAAGGTCGGTGTGAACG |
|  | Reverse: CTCGCTCCTGGAAGATGGTG |
